# Supplementary material for: Modeling glioblastoma heterogeneity as a dynamic network of cell states
Source: Mol Syst Biol. 2021 Sep 16;17(9):e10105. doi: 10.15252/msb.202010105 (PMC8444284; doi:10.15252/msb.202010105)
Supplement: Supplementary file 6 — Source Data for Figure 5 [file MSB-17-e10105-s004.zip › Figure5A_sourcedata/GSEA_3017/nosigdb_stateD.GseaPreranked.1622795010397/FELDMAN_2020_M-EARLYG1.html]

Details for gene set FELDMAN\_2020\_M-EARLYG1[GSEA]

|  || Dataset | state23017 |
| Phenotype | NoPhenotypeAvailable |
| Upregulated in class | na\_neg |
| GeneSet | FELDMAN\_2020\_M-EARLYG1 |
| Enrichment Score (ES) | -0.6512577 |
| Normalized Enrichment Score (NES) | -2.926334 |
| Nominal p-value | 0.0 |
| FDR q-value | 0.0 |
| FWER p-Value | 0.0 |
Table: GSEA Results Summary

  

Fig 1: Enrichment plot: FELDMAN\_2020\_M-EARLYG1      
 Profile of the Running ES Score & Positions of GeneSet Members on the Rank Ordered List

  

| PROBE | GENE SYMBOL | GENE\_TITLE | RANK IN GENE LIST | RANK METRIC SCORE | RUNNING ES | CORE ENRICHMENT || 1 | PTTG1 |  |  | 14 | 0.490 | 0.0077 | No |
| 2 | PHLDA1 |  |  | 59 | 0.336 | -0.0138 | No |
| 3 | LDHA |  |  | 92 | 0.297 | -0.0275 | No |
| 4 | TROAP |  |  | 109 | 0.283 | -0.0292 | No |
| 5 | LSM5 |  |  | 123 | 0.274 | -0.0289 | No |
| 6 | BIRC5 |  |  | 130 | 0.271 | -0.0233 | No |
| 7 | PLD3 |  |  | 240 | -0.258 | -0.0985 | No |
| 8 | FTL |  |  | 271 | -0.261 | -0.1120 | No |
| 9 | GPM6B |  |  | 314 | -0.265 | -0.1347 | No |
| 10 | GLIPR2 |  |  | 411 | -0.275 | -0.1992 | No |
| 11 | CKB |  |  | 554 | -0.292 | -0.2990 | No |
| 12 | CYR61 |  |  | 555 | -0.292 | -0.2878 | No |
| 13 | S100B |  |  | 705 | -0.320 | -0.3920 | No |
| 14 | IGFBP7 |  |  | 733 | -0.326 | -0.4006 | No |
| 15 | MEST |  |  | 864 | -0.360 | -0.4884 | No |
| 16 | TUBB4B |  |  | 916 | -0.380 | -0.5137 | No |
| 17 | MOXD1 |  |  | 996 | -0.412 | -0.5597 | No |
| 18 | SAT1 |  |  | 1109 | -0.473 | -0.6292 | No |
| 19 | SPARC |  |  | 1127 | -0.493 | -0.6236 | No |
| 20 | TTYH1 |  |  | 1144 | -0.505 | -0.6169 | No |
| 21 | ASPM |  |  | 1189 | -0.567 | -0.6297 | Yes |
| 22 | FABP7 |  |  | 1190 | -0.568 | -0.6080 | Yes |
| 23 | CLU |  |  | 1207 | -0.592 | -0.5980 | Yes |
| 24 | CST3 |  |  | 1209 | -0.595 | -0.5761 | Yes |
| 25 | BEX1 |  |  | 1216 | -0.615 | -0.5573 | Yes |
| 26 | SERPINE1 |  |  | 1218 | -0.617 | -0.5346 | Yes |
| 27 | CKS2 |  |  | 1226 | -0.641 | -0.5156 | Yes |
| 28 | CTGF |  |  | 1234 | -0.654 | -0.4962 | Yes |
| 29 | CENPA |  |  | 1243 | -0.679 | -0.4766 | Yes |
| 30 | CCNA2 |  |  | 1255 | -0.716 | -0.4579 | Yes |
| 31 | UBE2T |  |  | 1256 | -0.719 | -0.4305 | Yes |
| 32 | HMGB2 |  |  | 1263 | -0.736 | -0.4071 | Yes |
| 33 | AURKA |  |  | 1271 | -0.763 | -0.3835 | Yes |
| 34 | MALAT1 |  |  | 1289 | -0.884 | -0.3631 | Yes |
| 35 | KPNA2 |  |  | 1297 | -0.972 | -0.3316 | Yes |
| 36 | CDK1 |  |  | 1311 | -1.433 | -0.2871 | Yes |
| 37 | UBE2C |  |  | 1312 | -1.466 | -0.2313 | Yes |
| 38 | TOP2A |  |  | 1313 | -1.528 | -0.1731 | Yes |
| 39 | CCL2 |  |  | 1317 | -1.880 | -0.1039 | Yes |
| 40 | HIST1H4C |  |  | 1320 | -2.769 | -0.0000 | Yes |
Table: GSEA details [plain text format]

  

Fig 2: FELDMAN\_2020\_M-EARLYG1: Random ES distribution      
 Gene set null distribution of ES for **FELDMAN\_2020\_M-EARLYG1**
